# Human-pathogenic relapsing fever *Borrelia* found in bats from Central China phylogenetically clustered together with relapsing fever borreliae reported in the New World

**DOI:** 10.1371/journal.pntd.0009113

**Published:** 2021-03-18

**Authors:** Ze-Min Li, Xiao Xiao, Chuan-Min Zhou, Jian-Xiao Liu, Xiao-Lan Gu, Li-Zhu Fang, Bin-Yan Liu, Lian-Rong Wang, Xue-Jie Yu, Hui-Ju Han

**Affiliations:** 1 State Key Laboratory of Virology, School of Health Sciences, Wuhan University, Wuhan, Hubei, China; 2 Institute of Epidemiology Research, Hubei University of Chinese Medicine, Wuhan, Hubei, China; 3 Clinical Laboratory, Xingtai Third Hospital, Xingtai, Hebei, China; 4 Key Laboratory of Combinatorial Biosynthesis and Drug Discovery, Ministry of Education, School of Pharmaceutical Sciences, Wuhan University, Wuhan, Hubei, China; Baylor College of Medicine, UNITED STATES

## Abstract

Bats can harbor zoonotic pathogens causing emerging infectious diseases, but their status as hosts for bacteria is limited. We aimed to investigate the distribution, prevalence and genetic diversity of *Borrelia* in bats and bat ticks in Hubei Province, China, which will give us a better understanding of the risk of *Borrelia* infection posed by bats and their ticks. During 2018–2020, 403 bats were captured from caves in Hubei Province, China, 2 bats were PCR-positive for *Borrelia*. Sequence analysis of *rrs*, *flaB* and *glpQ* genes of positive samples showed 99.55%-100% similarity to *Candidatus* Borrelia fainii, a novel human-pathogenic relapsing fever *Borrelia* species recently reported in Zambia, Africa and Eastern China, which was clustered together with relapsing fever *Borrelia* species traditionally reported only in the New World. Multilocus sequence typing (MLST) and pairwise genetic distances further confirmed the *Borrelia* species in the bats from Central China as *Candidatus* Borrelia fainii. No *Borrelia* DNA was detected in ticks collected from bats. The detection of this human-pathogenic relapsing fever *Borrelia* in bats suggests a wide distribution of this novel relapsing fever *Borrelia* species in China, which may pose a threat to public health in China.

## Author summary

Bats have a wide range of species and distribution, these mammals have been proposed as the natural reservoirs of pathogens for many years. Previous research focus mainly on viral agents in bats, while the infection of *Borrelia* spirochetes in bats is much neglected. *Borrelia* species causing Lyme disease and relapsing fever have been found in a wide range of ticks. Relapsing fever has long been recognized as major cause of disease and death in Africa, but as more and more cases reported in different continents, relapsing fever has become an emergent disease. There is growing evidence that bats can play a role in carrying *Borrelia* species. In this study, we captured different species of bats from caves during 2018–2020 in Central China, tested liver tissue for *Borrelia* by PCR, and applied multilocus sequence typing (MLST) to identify this bacterium. Our study found human pathogenic relapsing fever *Candidatus* Borrelia fainii in Central China, suggesting bat borne Borrelia spirochetes may pose a threat to public health in China.

## Introduction

Relapsing fever is a zoonosis caused by the relapsing fever group spirochetes of the genus *Borrelia*, and its clinical symptoms include recurrent febrile episodes with headache, myalgia, chills, and nausea [1]. To date, 27 relapsing fever *Borrelia* species spirochetes have been identified [2], but additional species have been proposed. Typically, relapsing fever borreliae were thought to be transmitted by soft ticks, with the exception of the human louse-borne *B*. *recurrentis* as well as the hard tick-borne *B*. *lonestari* and *B*. *miyamotoi* [3–5]. Historically, the relapsing fever borreliae were classified by the “one vector one species” concept, and in each region, a specific relationship existed between soft ticks and *Borrelia* spp. [6]. According to the epidemic areas and genetic lineage of the causative agent, the relapsing fever borreliae were rather arbitrarily divided into two subgroups: the Old World borreliae (the Palearctic and Afrotropic ecozone) and the New World borreliae (Nearctic ecozone) [7].

Bats have attracted much attention from their close relationship with emerging viral infectious diseases, including Nipah, Hendra, SARS, MERS and the recent worldwide pandemic COVID-19 [8]. It is worth noting that previous research focused mainly on viral agents in bats, while the infection and prevalence of bacteria in bats are much neglected [9]. Knowing whether bats can act as carriers of pathogenic bacteria is a common interest for public health [10–12]. Ticks, as one of the parasites of bats, act as the most important arthropod vectors for transmitting microbial pathogens to animals and humans. Once bitten by these ticks, the risk of infection will increase. Therefore, the role of both bats and their ticks in carrying or transmitting bacteria deserves more study. A recent study found bats and bat soft ticks from a cave in Zambia, Africa showed a high infection rate with *Candidatus* Borrelia fainii, which was a novel human-pathogenic relapsing fever *Borrelia* species clustered together with the New World relapsing fever borreliae. It proposed that bats and bat soft ticks contributed to the environmental cycle of this *Borrelia* species as hosts and vectors, respectively [13]. Our group also found that one *Myotis* bat from Shandong Province in Eastern China was infected with *Candidatus* Borrelia fainii, as the first report of this novel relapsing fever *Borrelia* species in Asia [14]. Since only a single bat was found to be infected with the *Borrelia* species, the role of bats in the transmission of *Borrelia* cannot be determined in China. In this study, we aimed to investigate the distribution, prevalence and genetic diversity of *Borrelia* in bats and bat ticks from Hubei Province, China, which will give us a better understanding of the risk of *Borrelia* infection posed by bats and their ticks.

## Material and methods

### Ethical statement

The collection of bats for microbiological studies was approved by the Ethics Committee of the Medical School, Wuhan University (WHU2020-YF0023), and all efforts were made to minimize discomfort to the animals.

### Bat sampling

**Fig 1:**
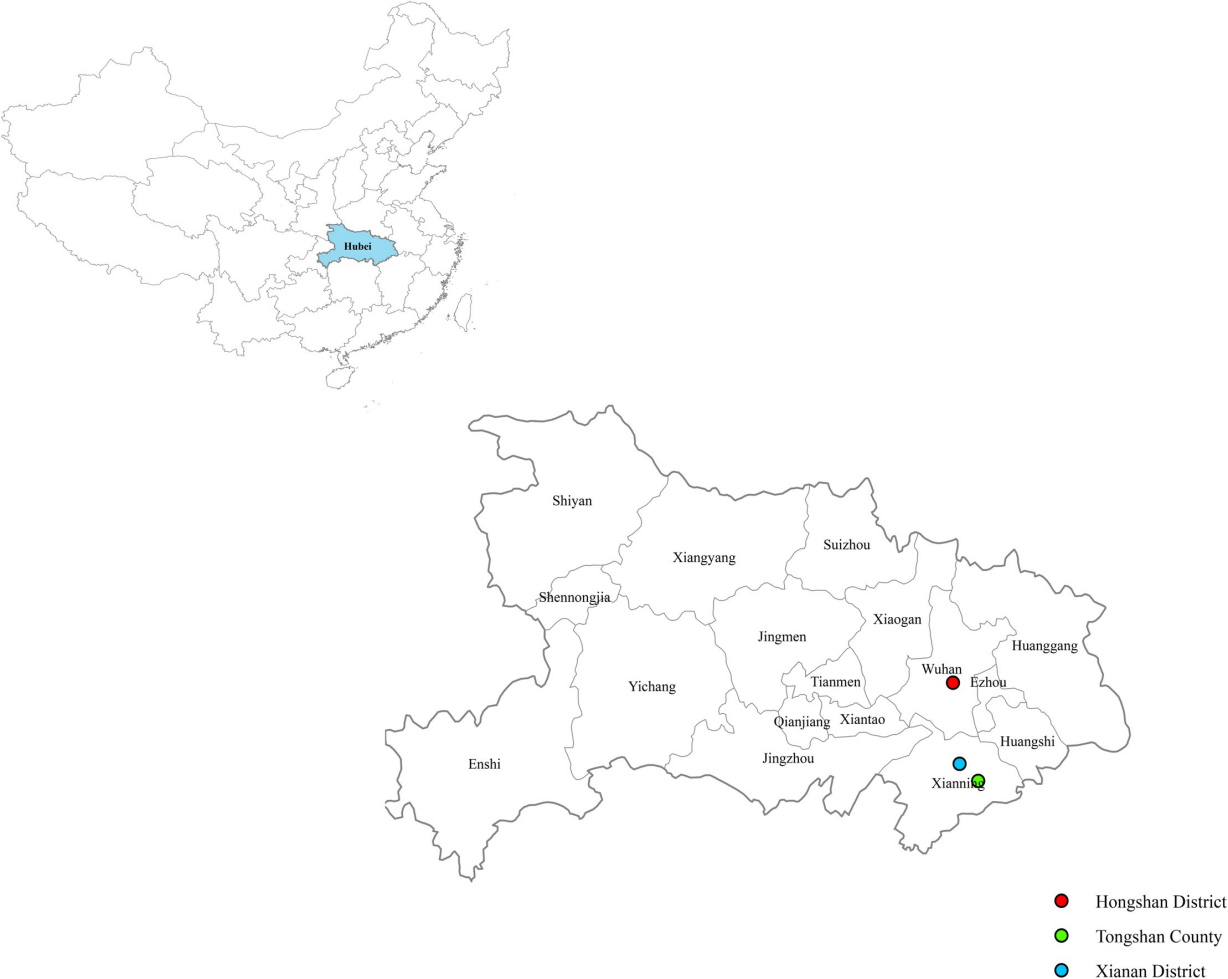
Map showing the location where bats were collected in Hubei Province, China. The map constructed using ArcGIS 10.6 software.

From May 2018 to August 2020, bats were collected from caves in Xianning City and Wuhan City of Hubei Province in Central China (Fig 1). Bats were captured using mist nets settled near the entrance of the cave at sunset when bats left for night feeding, and bats were collected early the next morning. Bats were euthanized by an overdose of chloral hydrate administered via intraperitoneal injection, and tissue samples were collected and stored at -80°C. Bat species were identified with morphology, and then confirmed by amplifying and sequencing the cytochrome B (*cytB*) gene as described previously [15].

### Molecular detection of relapsing fever borreliae in bats

DNA was extracted from the liver tissue of each bat with QIAGEN DNA Kit (Qiagen, Valencia, CA) according to the manufacturer’s instructions. Bats were initially screened with the universal 16S rRNA (*rrs*) primers of bacteria [16], and two bats were found positive for relapsing fever *Borrelia*. To further characterize the relapsing fever *Borrelia* from the bats, *Borrelia* flagellin (*flaB*) [17] and the glycerophosphodiester phosphodiesterase (*glpQ*) [18] genes were amplified (S1 Table) with PCR.

The PCR reaction was conducted in a 15 μL mixture containing 7.5 μL of 2X Taq Master Mix (Vazyme Biotech, China), 0.5 μL of 10 μM of each forward and reverse primer (Sangon Biotech, Shanghai, China), 4.5 μL of nuclease-free water, and 2 μL liver DNA of each sample. Nuclease-free water was used as a negative control. To prevent contamination, no positive control was used in the study. PCR was performed under the following conditions: 1 denaturing cycle at 95°C for 5 min followed by 40 cycles at 95°C for 30 s, 55°C for 30 s, and 72°C for 90 s, and an additional final cycle at 72°C for 10 min. Each PCR assay included a negative control (distilled water instead of DNA template).PCR products were analyzed with 1.2% agarose gel electrophoresis, and bands of expected size (1,494 bp for *rrs*, 753 bp for *flaB*, 599 bp for *glpQ* fragment 1, and 453 bp for *glpQ* fragment 2) were excised from gels, and purified with a Gel Extraction Kit (TSINGKE Biological Technology, China). The purified amplicons were inserted into the pMD19-T vector (TaKaRa, Shiga, Japan) for cloning, and at least three positive clones were selected for sequencing with universal primers M13-47 and M13-48. Chromatograms were checked with Chromas 2.5.1 (Technelysium, Tewantin, QLD) to ensure the accuracy of sequencing. The 2 *glpQ* fragments were assembled into an 880 bp sequence with Lasergene 7.1 (DNASTAR, Madison, WI, USA). The obtained sequences were compared to those in public databases using the Nucleotide Basic Local Alignment Search Tool (BLASTn) on the National Center for Biotechnology Information website (http://blast.ncbi.nlm.nih.gov/Blast.cgi).

### Molecular detection of relapsing fever borreliae in bat ticks

Ticks were collected from bats, and tick species were identified by PCR targeting tick mitochondrial 16S rDNA as previously described [19] (S1 Table). DNA was extracted from pools of ticks with AllPrep DNA/RNA Mini Kit (Qiagen, Valencia, CA). Examination of the *Borrelia* DNA in the ticks was performed by PCR and the sequencing of amplicons as described above.

### Phylogenetic analysis

Phylogenetic analysis was conducted using MEGA 7.0. ClustalW software to align the sequences. Phylogenetic trees were constructed based on neighbor-joining method with the Kimura 2-parameter model in MEGA 7.0, bootstrap values were calculated with 1, 000 replicates.

### Multilocus sequence typing of relapsing fever borreliae

For relapsing fever *Borrelia*-positive samples, MLST was attempted by PCR amplification of 8 housekeeping genes (*clpA*, *clpX*, *nifS*, *pepX*, *pyrG*, *recG*, *rplB* and *uvrA*) with degenerate primers kindly provided by Dr. Gabriele Margos, who is the curator of the *Borrelia* MLST database (https://pubmlst.org/borrelia/) (S1 Table). The amplicons were cloned for sequencing as described above.

Sequences were analyzed using the sequence query function of the *Borrelia* MLST database, and each gene was compared with its existing alleles. If the sequence was new to the database, new allele numbers were assigned after being submitted to the *Borrelia* MLST database, and based on the allelic profile of the 8 housekeeping genes, the *Borrelia* species would be assigned an existing or new (for those with new combinations of alleles or novel alleles) ST number.

Sequences of the 8 housekeeping genes were trimmed as described in the *Borrelia* MLST database, and concatenated in the order of *clpA*, *clpX*, *nifS*, *pepX*, *pyrG*, *recG*, *rplB* and *uvrA*. Concatenated sequences were imported into MEGA 7.0. After being aligned with ClustalW, a phylogenetic tree was constructed by using the neighbor-joining method with the Kimura 2-parameter model in MEGA 7.0.

Pairwise genetic distances were calculated by using the Kimura-2 model, and relapsing fever *Borrelia* species were identified using the threshold (98.3% similarity, genetic distance 0.017) [20]. Reference sequences of *Borrelia* species included in the analyses were obtained from the *Borrelia* MLST database as well as the GenBank.

## Results

### Bat and tick sampling

From May 2018 to August 2020, a total of 403 bats were sampled from caves in Xianning City and Wuhan City, Hubei Province, China. By amplifying and sequencing the *cytB* gene, bats were identified into five species of two families (Table 1). Fourteen ticks were collected from bats sampled in July 2020, and they were identified as 8 *Carios vespertilionis* and 6 *Ixodes simplex*. The *cytB* gene of representative bat species and the 16S rDNA sequences of bat ticks were deposited in GenBank with accession No. MH888178, MH888180, MW085077-MW085079 and MW132810-MW132811.

**Table 1:** Summary of bat sampling information.

Sampling date	Sampling area	Bat family	Bat species	Bats No.
May 2018	Xianan District, Xianning (29°78′N, 114°31′E)	Vespertilionidae	*Myotis davidii*	42
			*Myotis adversus*	15
			*Myotis altarium*	2
			*Miniipterus schreibersii*	9
July 2020	Tongshan County, Xianning (29°61′N, 114°48′E)	Vespertilionidae	*Myotis davidii*	124
			*Myotis adversus*	38
		Rhinolophidae	*Rhinolophus pusillus*	154
August 2020	Hongshan District, Wuhan (30°50′N, 114°34′E)	Vespertilionidae	*Myotis davidii*	16
			*Myotis adversus*	1
			*Myotis altarium*	1
		Rhinolophidae	*Rhinolophus pusillus*	1
**Total**				**403**

### Molecular detection of relapsing fever borreliae

Two out of 403 bats were positive for relapsing fever *Borrelia* by PCR amplification of *rrs*, *flaB* and *glpQ*. The 2 positive bats were *Rh*. *pusillus* (bat ID: XN788) and *My*. *davidii* (bat ID: XN888) collected from Xianning City in July 2020. *Borrelia* DNA was also detected in the spleen, lung, kidney and blood samples of *Borrelia*-positive bats. Tick was not found on these two infected bats, and no *Borrelia* DNA was detected in ticks collected from bats.

**Fig 2:**
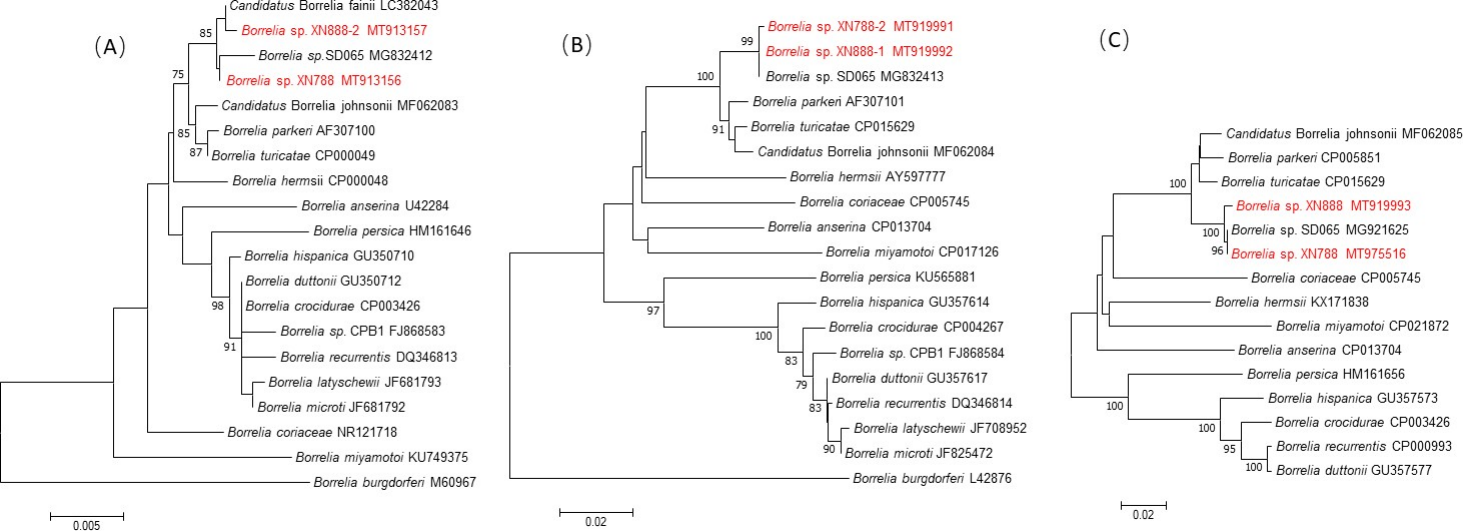
Phylogenetic analysis of (A) *rrs*, (B) *flaB* and (C) *glpQ* gene of relapsing fever *Borrelia* species. The sequences M60967 from (A) and L42876 from (B) are outgroup. Phylogenetic tree was drawn using the neighbor-joining method with the Kimura 2-parameter model in MEGA 7.0. Phylogenetic tree representing the relationships among *Borrelia* identified in this study (50% boot cutoff). Bootstrap values are indicated at the nodes. Scale bar indicates the degree of divergence represented by a given length of branch. Boldface indicates the taxonomic position of *Borrelia*. *Borrelia* species identified in bats from this study were shown in red.

Based on *rrs*, *flaB* and *glpQ* genes, relapsing fever *Borrelia* detected in bats from this study were closely related to *Borrelia* sp. Qtaro and *Borrelia* sp. SD065, which were designated as *Candidatus* Borrelia fainii, and were reported in Zambia and China, respectively (Table 2). Neighbor-joining phylogenetic trees constructed based on *rrs*, *flaB* and *glpQ* genes showed that *Borrelia* detected in bats of this study clustered with a group of human pathogenic New World relapsing fever borreliae (Fig 2). The *rrs*, *flaB* and *glpQ* genes of relapsing fever *Borrelia* of this study were deposited in GenBank with accession No. MT913156-MT913157 (*rrs*), MT919991-MT919992 (*flaB*) and MT975516, MT919993 (*glpQ*).

**Table 2:** Best matches to *Borrelia* gene sequences found in GenBank.

*Borrelia*-positive bat ID number	Bat species	*Borrelia* gene	Closest match in GenBank	Accession number of closest match	Nucleotide identify	Accession number of this study
XN788	*Rhinolophus pusillus*	*rrs*	*Borrelia* sp. Qtaro	LC382043	99.93% (1493/1494)	MT913156
		*flaB*	*Borrelia* sp. SD065	MG832413	99.86% (750/751)	MT919991
		*glpQ*	*Borrelia* sp. SD065	MG921625	100%(880/880)	MT975516
XN888	*Myotis davidii*	*rrs*	*Borrelia* sp. Qtaro	LC382043	99.93% (1493/1494)	MT913157
		*flaB*	*Borrelia* sp. SD065	MG832413	100% (751/751)	MT919992
		*glpQ*	*Borrelia* sp. SD065	MG921625	99.55% (876/880)	MT919993

### MLST analysis

**Fig 3:**
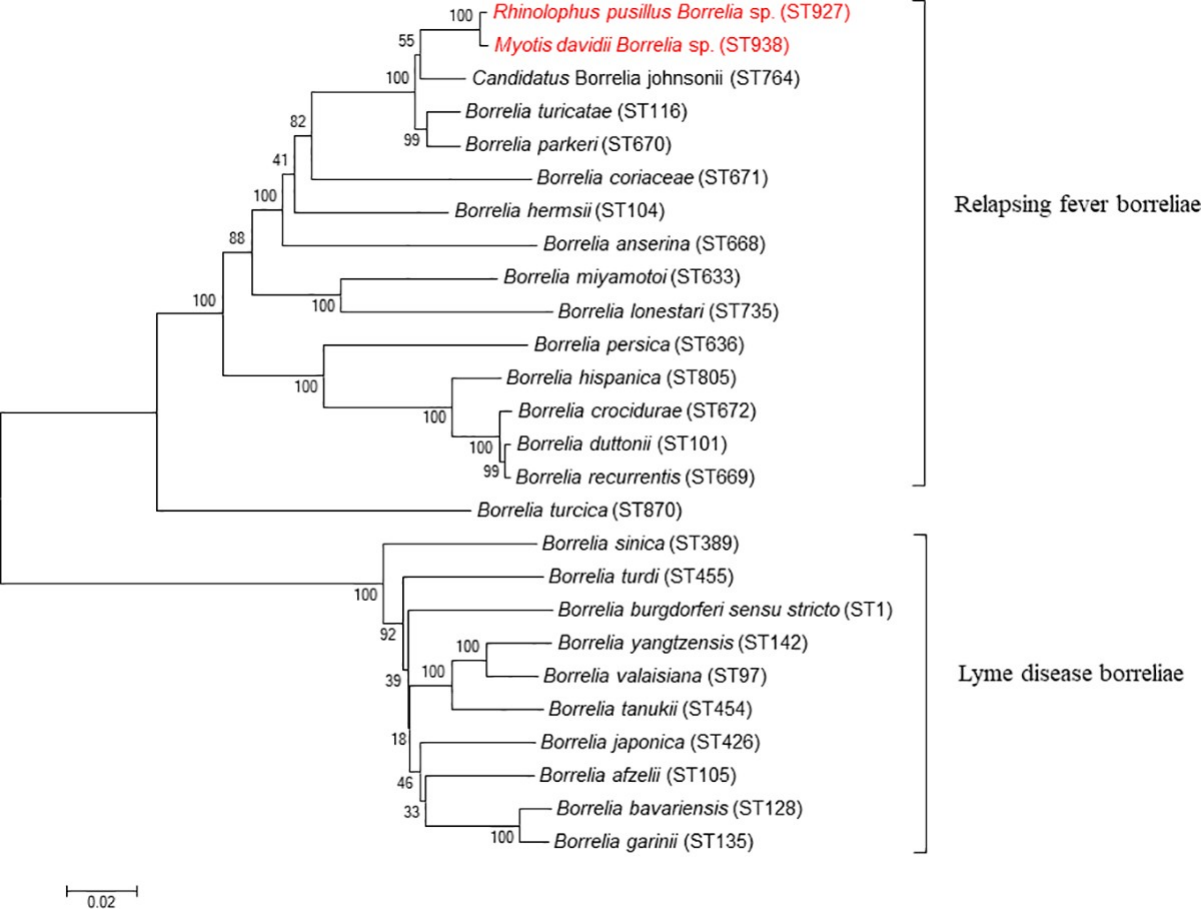
Phylogenetic analysis of borreliae based on concatenated sequences of the 8 loci in the order of *clpA*, *clpX*, *nifS*, *pepX*, *pyrG*, *recG*, *rplB* and *uvrA*. Phylogenetic tree was drawn using the neighbor-joining method with the Kimura 2-parameter model in MEGA 7.0. Phylogenetic tree representing the relationships among *Borrelia* identified in this study (50% boot cutoff). Bootstrap values are indicated at the nodes. Scale bar indicates the degree of divergence represented by a given length of branch. Boldface indicates the taxonomic position of *Borrelia*. *Borrelia* sequences identified in bats from this study were shown in red.

For *Borrelia*-positive *Rh*. *pusillus* (bat ID: XN788), sequence query showed that the 8 housekeeping genes were exactly the same as ST927, which was previously reported by our team in a *Myotis ricketti* bat from Shandong Province, China [14]. For *Borrelia*-positive *My*. *davidii* (bat ID: XN888), the 8 loci of MLST were all novel alleles, and the 8 loci were submitted to the *Borrelia* MLST database and novel allele numbers were assigned: *clpA* (303), *clpX* (263), *nifS* (238), *pepX* (267), *pyrG* (278), *recG* (294), *rplB* (256), and *uvrA* (270), and sequence typing was assigned as ST938. Phylogenetic analysis based on the concatenated 8 housekeeping genes (4,776 bp, in the order of *clpA*-*clpX*-*nifS*-*pepX*-*pyrG*-*recG*-*rplB*-*uvrA*) revealed that the *Borrelia* species found in bats from this study clustered with a group of human pathogenic relapsing fever *Borrelia* spirochetes, and were most related to *Candidatus* Borrelia fainii, which was recently identified in a febrile patient, bats and soft ticks from Zambia [13], as well as in a bat from Shandong Province, China [14] (Fig 3). Phylogenetic trees based on each of the 8 loci of relapsing fever *Borrelia* were shown in S1–S8 Figs. Genetic distance analysis of the concatenated 8 loci (4,776 bp) revealed a value of 0.015 compared to *Candidatus* Borrelia fainii strain Qtar (S2 Table). Herein, the *Borrelia* species detected in bats from this study was identified as *Candidatus* Borrelia fainii.

## Discussion

In this study, we found that two bats captured from Xianning City, Hubei Province of China were positive for *Candidatus* Borrelia fainii, which was recently isolated from a patient diagnosed with relapsing fever in Zambia, and clustered together with the relapsing fever borreliae [13]. *Candidatus* Borrelia fainii is closely related to a group of New World relapsing fever borreliae, including *B*. *turicatae*, *B*. *parkeri* and *Candidatus* Borrelia johnsonii, which were endemic in USA. *Borrelia turicatae* and *B*. *parkeri* cause tick-borne relapsing fever in humans[21,22], and they are predominantly prevalent in the southwestern of USA [23]. *Candidatus* Borrelia johnsonii was firstly reported in bat soft ticks distributed in the USA [24]. A recent study found that *Candidatus* Borrelia johnsonii was associated with tick-borne diseases in humans [20].

Previous reports of *Borrelia* species in bats were only individual cases [25,26]. A novel Old World relapsing fever *Borrelia* species (CPB1) was found responsible for fatal borreliosis in a *Pipistrelle* bat from the UK [25]. Subsequently, *Borrelia* species CPB1 was detected in soft ticks from bats in France [27], indicating the association of relapsing fever *Borrelia* spirochetes and bat soft ticks. A recent study found that bats and bat soft ticks collected from a cave in Zambia showed a high infection rate for *Candidatus* Borrelia fainii, and proposed that bats and bat soft ticks contributed to the environmental cycle of *Candidatus* Borrelia fainii as hosts and vectors, respectively [13]. However, our previous study found that only one out of 145 bats collected from Shandong Province, China was infected with *Candidatus* Borrelia fainii [14]. In this study, only 2 out of 403 bats were found positive for *Candidatus* Borrelia fainii. The *Borrelia*-positive bat species found in China in this study include *Myotis ricketti* in Shandong Province [14], *Rhinolophus pusillus* and *Myotis davidii* in Hubei Province of this study. *Myotis ricketti* is distributed in China, Vietnam and Laos, *Rhinolophus pusillus* can be found in Southern China, India, Nepal, Myanmar, Vietnam, Thailand, Malaysia and Indonesia, while *Myotis davidii* is endemic to China (http://www.bio.bris.ac.uk/research/bats/China bats/). Besides, the liver and spleen of the two *Borrelia*-positive bats appeared to be enlarged. *Borrelia* DNA was also detected in spleen, lung, and kidney tissue as well as blood samples of *Borrelia*-positive bats, indicating multiorgan infection resulted from spirochetemia. However none of the ticks collected from these bats was positive for *Borrelia* by PCR, possibly due to the small sample size. Given the facts that this relapsing fever *Borrelia* species infection could be fatal to bats and the infection rate of this *Borrelia* species in bats was low, *Borrelia* infection in bats might be incidental events, just as the cases in humans. Further studies are needed to investigate the reservoir and vector of this novel relapsing fever *Borrelia* species in China.

Besides *Candidatus* Borrelia fainii, there were also several reports of the relapsing fever *Borrelia* found in Africa that phylogenetically clustered together with relapsing fever borreliae reported in the New World. A new human pathogenic *Borrelia* species was identified in *Ornithodoros* ticks from Tanzania, and it grouped together with the relapsing fever borreliae reported in the New World rather than the relapsing fever-inducing spirochetes that were known to be endemic in East Africa [11,28]. Another two studies described the discovery of a novel relapsing fever *Borrelia*, *Candidatus* Borrelia kalaharica, in travelers returning from the Kalahari Desert, South Africa [29,30].

The presence of *Candidatus* Borrelia fainii in Zambia, as well as the discovery of *Borrelia* species (*B*. *lonestari* and *B*. *miyamotoi*) that were related to relapsing fever borreliae but were transmitted by hard ticks rather than soft ticks challenged previous taxonomies based largely on microbe-vector specificity and geographic distribution. With more and more studies, we might have a better understanding as to the classification of relapsing fever borreliae.

Conclusively, our studies found that the human pathogenic relapsing fever *Candidatus* Borrelia fainii in a new location in China and further study is needed to determine its true distribution in China. However, with the emergence of this human-pathogenic relapsing fever *Borrelia* species in China, it’s important to find the reservoir and vector of it.

## Supporting information

S1 Fig Phylogenetic analysis of relapsing fever borreliae based on *clpA* locus. (TIF)

S2 Fig Phylogenetic analysis of relapsing fever borreliae based on *clpX* locus. (TIF)

S3 Fig Phylogenetic analysis of relapsing fever borreliae based on *nifS* locus. (TIF)

S4 Fig Phylogenetic analysis of relapsing fever borreliae based on *pepX* locus. (TIF)

S5 Fig Phylogenetic analysis of relapsing fever borreliae based on *pyrG* locus. (TIF)

S6 Fig Phylogenetic analysis of relapsing fever borreliae based on *recG* locus. (TIF)

S7 Fig Phylogenetic analysis of relapsing fever borreliae based on *rplB* locus. (TIF)

S8 Fig Phylogenetic analysis of relapsing fever borreliae based on *uvrA* locus. Phylogenetic trees of S1–S8 Figs were drawn using the neighbor-joining method with the Kimura 2-parameter model with an alignment of the eight allelic sequences derived from the *Borrelia* MLST database (https://pubmlst.org/borrelia/) as well as from the GenBank. Corresponding GenBank number was shown in the brackets, and the sequences with * represents that the sequences were only submitted to the GenBank. Sequences amplified from this study were shown in red.
(TIF)

S1 Table PCR primers used in this study. (DOCX)

S2 Table Estimates of evolutionary divergence between relapsing fever *Borrelia* species based on the concatenated 8 housekeeping gene sequences (*clpA-clpX-nifS-pepX-pyrG-recG-rplB-uvrA*). (DOCX)
