# Supplementary figures and images for: Human-pathogenic relapsing fever Borrelia found in bats from Central China phylogenetically clustered together with relapsing fever borreliae reported in the New World
Source: PLoS Negl Trop Dis. 2021 Mar 18;15(3):e0009113. doi: 10.1371/journal.pntd.0009113 (PMC7971464; doi:10.1371/journal.pntd.0009113)

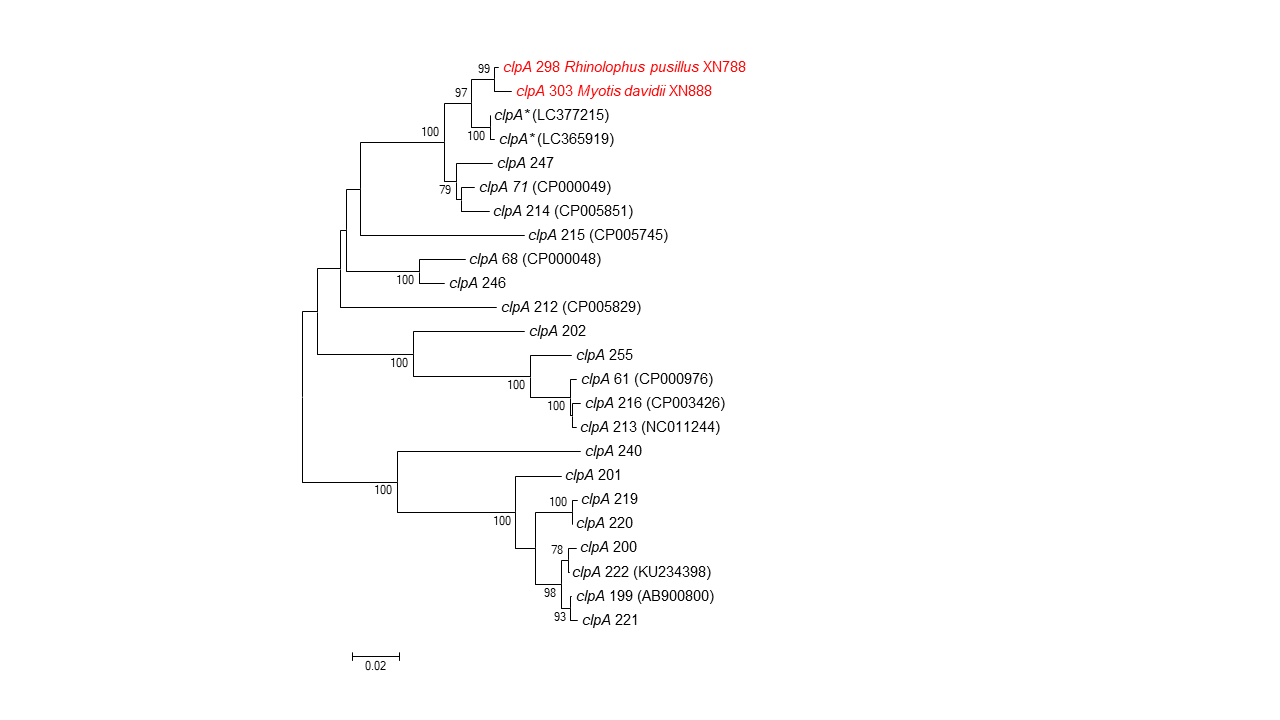

Supplement: S1 Fig — (TIF) [file pntd.0009113.s001.tif]

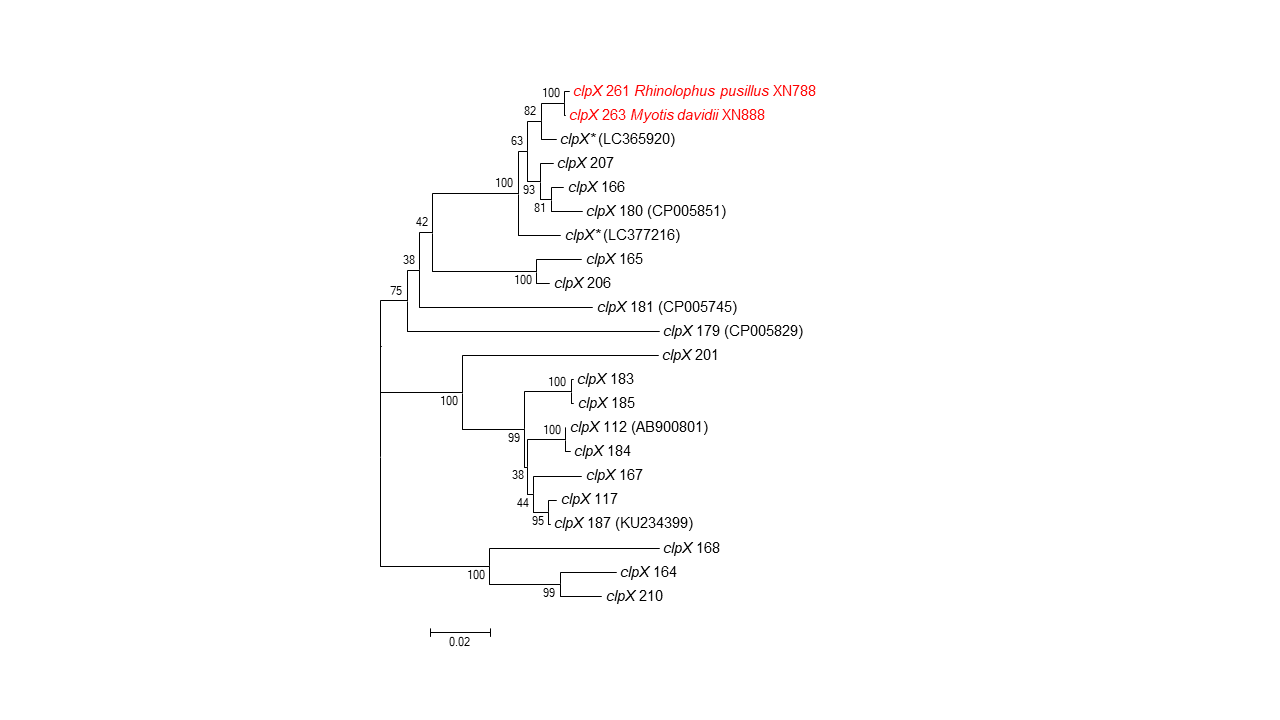

Supplement: S2 Fig — (TIF) [file pntd.0009113.s002.tif]

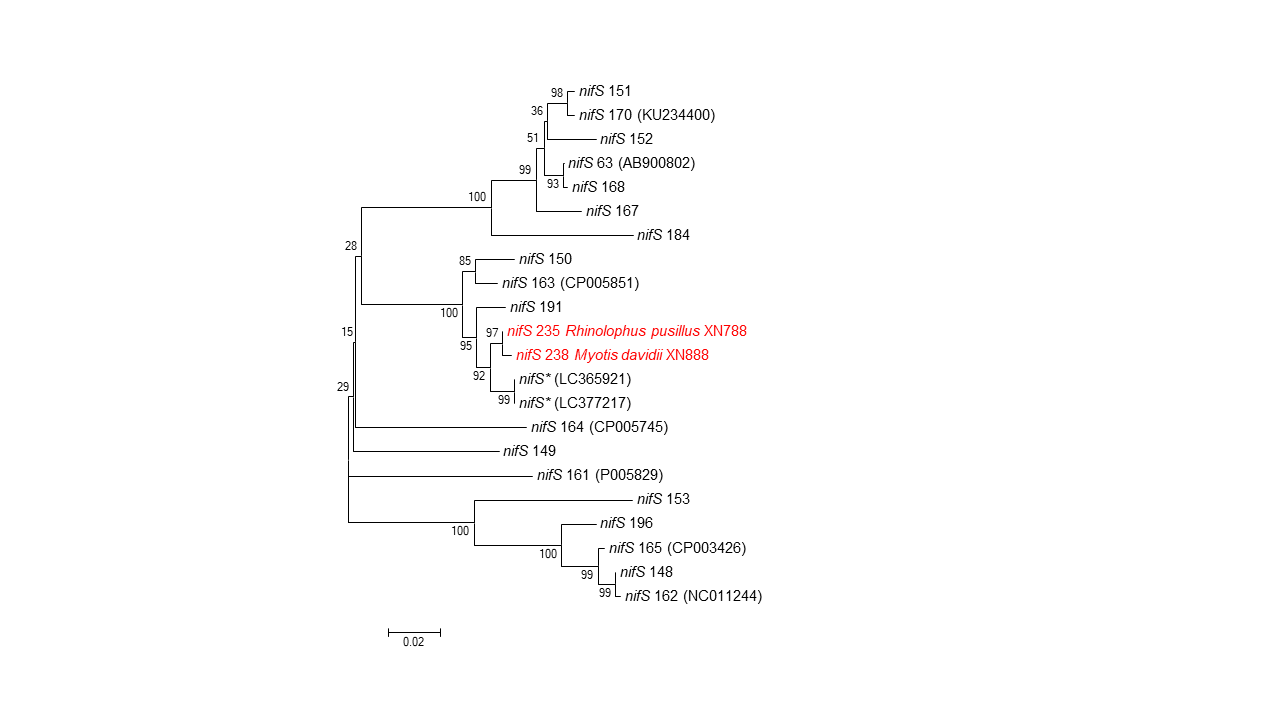

Supplement: S3 Fig — (TIF) [file pntd.0009113.s003.tif]

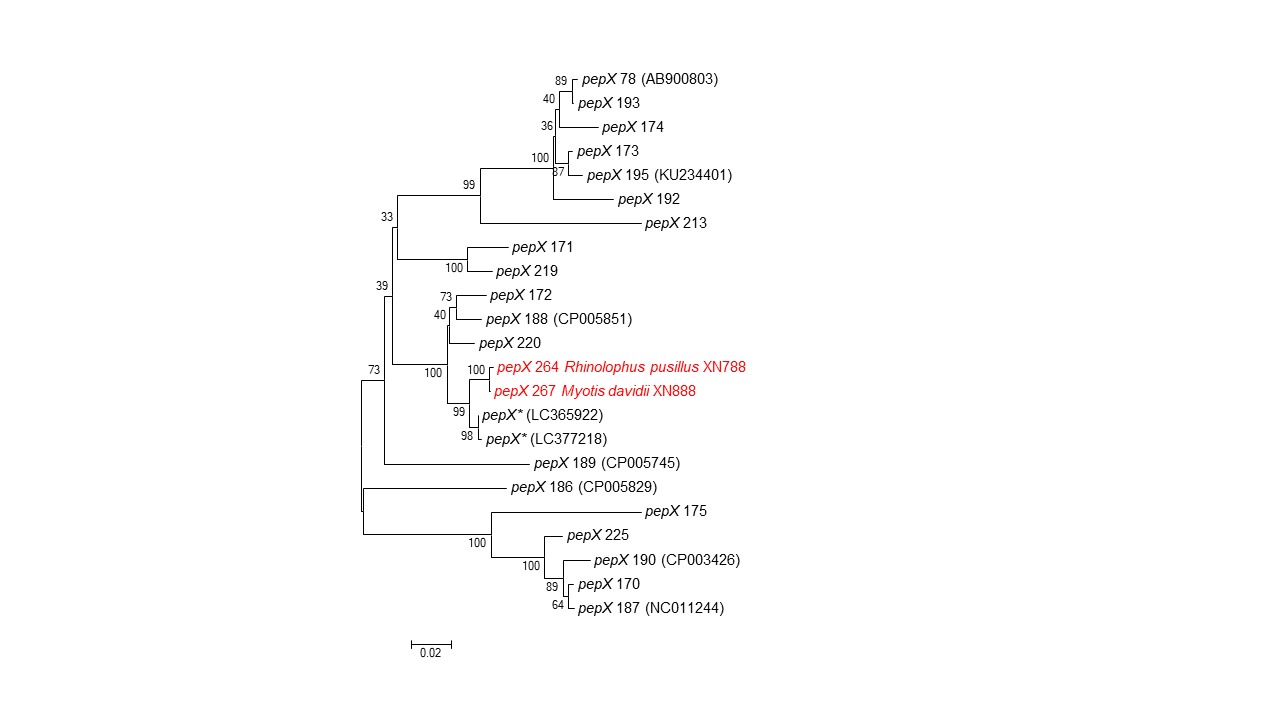

Supplement: S4 Fig — (TIF) [file pntd.0009113.s004.tif]

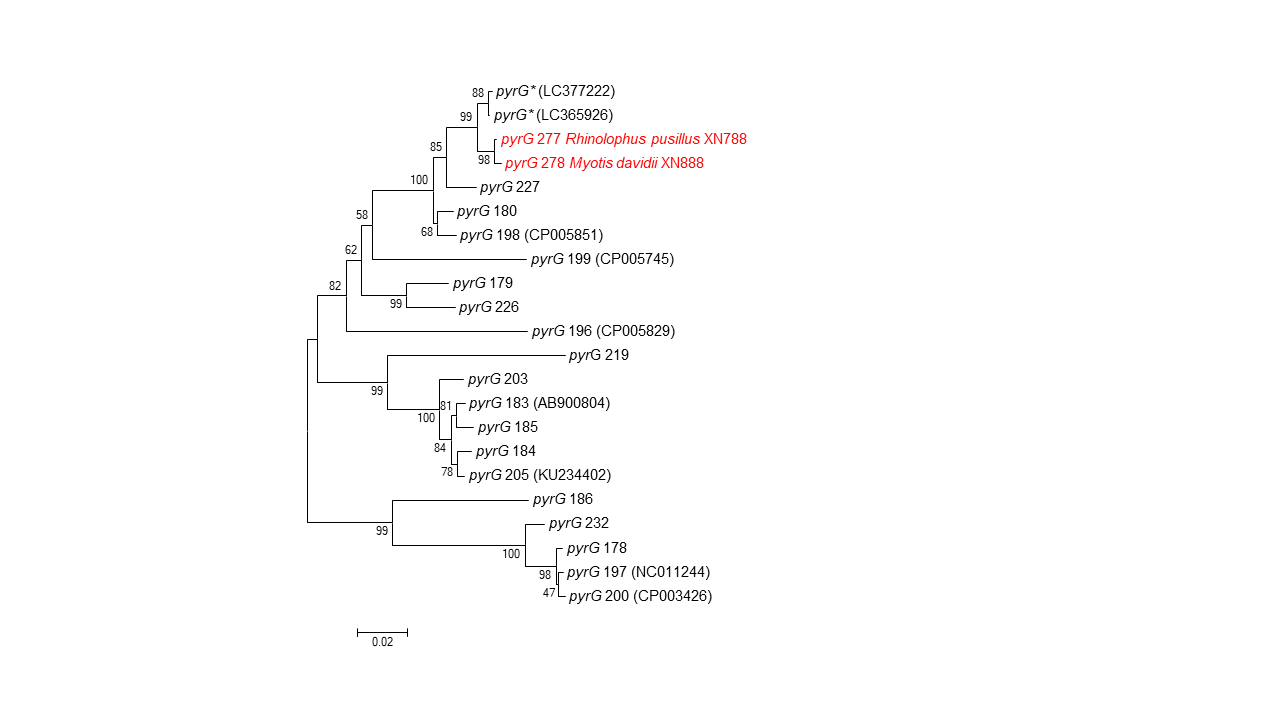

Supplement: S5 Fig — (TIF) [file pntd.0009113.s005.tif]

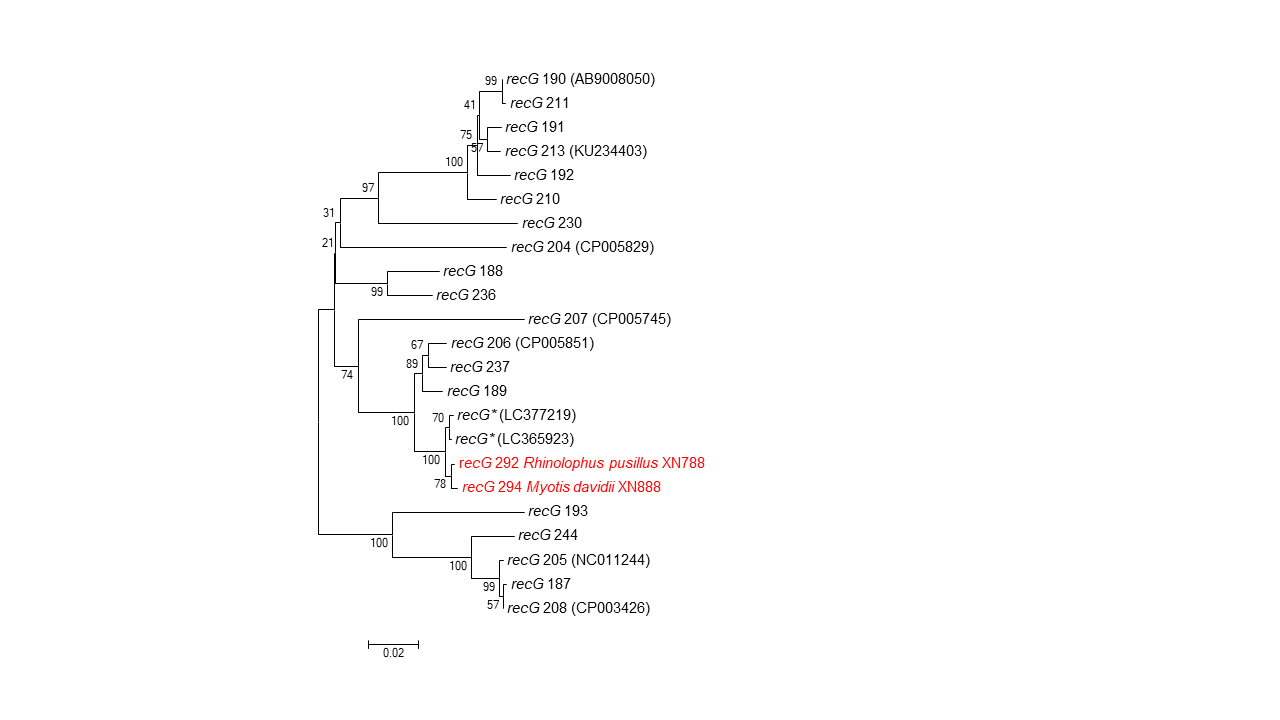

Supplement: S6 Fig — (TIF) [file pntd.0009113.s006.tif]

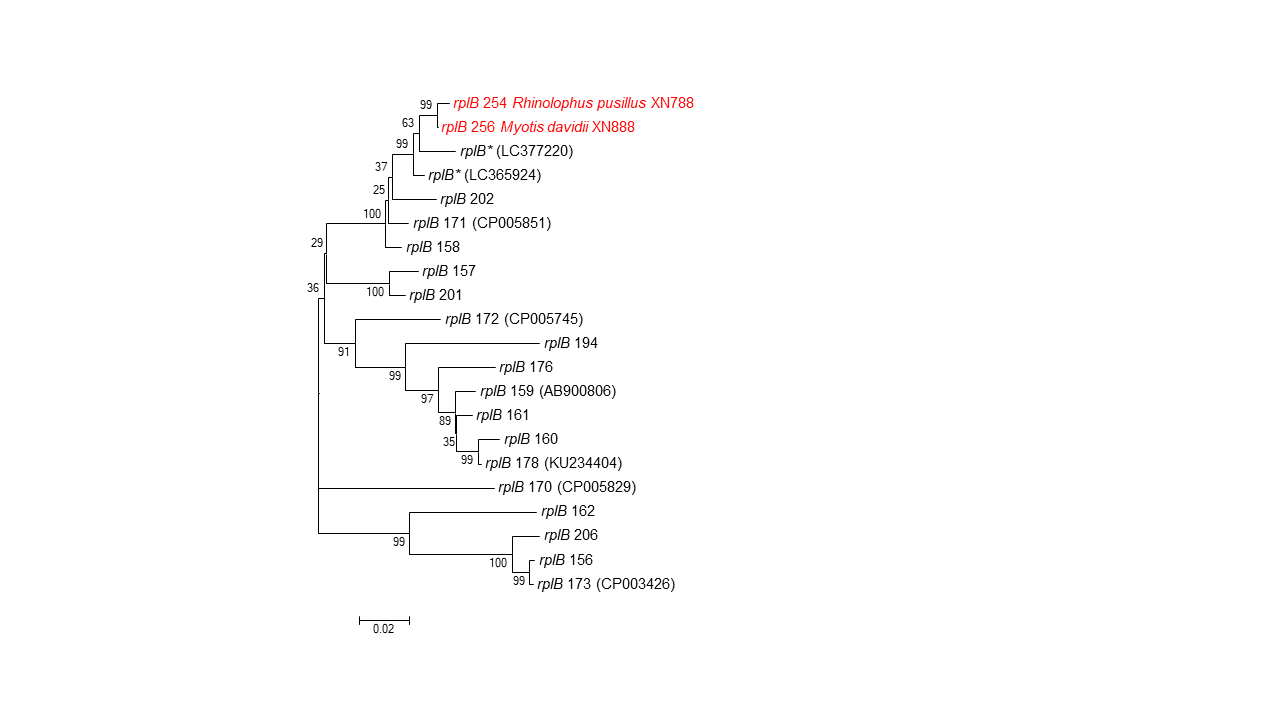

Supplement: S7 Fig — (TIF) [file pntd.0009113.s007.tif]

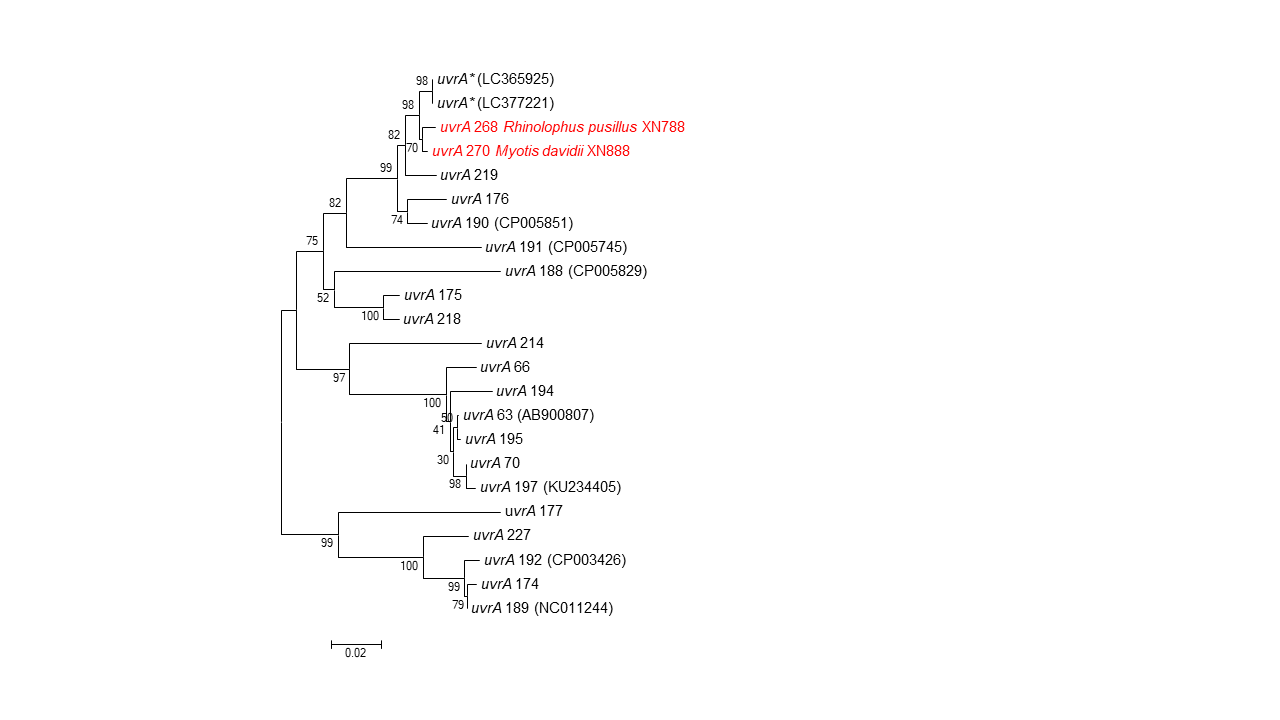

Supplement: S8 Fig — Phylogenetic trees of S1–S8 Figs were drawn using the neighbor-joining method with the Kimura 2-parameter model with an alignment of the eight allelic sequences derived from the Borrelia MLST database (https://pubmlst.org/borrelia/) as well as from the GenBank. Corresponding GenBank number was shown in the brackets, and the sequences with * represents that the sequences were only submitted to the GenBank. Sequences amplified from this study were shown in red. (TIF) [file pntd.0009113.s008.tif]
