# Supplementary material for: Human-pathogenic relapsing fever Borrelia found in bats from Central China phylogenetically clustered together with relapsing fever borreliae reported in the New World
Source: PLoS Negl Trop Dis. 2021 Mar 18;15(3):e0009113. doi: 10.1371/journal.pntd.0009113 (PMC7971464; doi:10.1371/journal.pntd.0009113)
Supplement: S1 Table — (DOCX) [file pntd.0009113.s009.docx]

**S1** **Table. PCR primers used in this study.**

| gene | primers | sequences (5′-3′ ) | PCR products (bp) | MLST locus  (bp) | PCR conditions | reference |
| --- | --- | --- | --- | --- | --- | --- |
| *clpX* | clpXF104 | CTGTTGCYATTTGTTTTGAATGYTC | 1,173 | 624^*^ | 9 cycles touchdown PCR:  94°C 30 s, annealing from 58°C-50°C  per cycle, 30 s, 72 °C 60 s, 35 cycles, 94°C 30 s, 50°C 30 s, 72 °C 60 s, a final extension: 72°C 5 min. | Primers provided by the curator of the *Borrelia* MLST database.^#^ |
|  | clpXR1183 | CTTTTTTAATTTGCTTASTWGAAGG |  |  |  |  |
| *pepX* | pepF361 | AGAGAYTTAAGYTTAKCAGG | 841 | 570 |  |  |
|  | pepR1187 | TGCATTCCCCACATTGG |  |  |  |  |
| *pyrG* | pyrF379 | TATTTAGGKAGAACTGTACAGC | 846 | 603^*^ |  |  |
|  | pyrR1375 | CAAGTCGCATTGTWGCAC |  |  |  |  |
| *recG* | recF898 | GCKTTTCTMTCTAGYATTCC | 871 | 651 |  |  |
|  | recR1779 | TTCRGTTAAAGGTTCCTTATAAAG |  |  |  |  |
| *rplB* | rplF3 | GGAGAAAAATATGGGKATTAAGAC | 743 | 624 |  |  |
|  | rplR759 | GATACAGGATGWCGACC |  |  |  |  |
| *uvrA* | uvrF1173 | GCGTTATCTTWCAACTGAATC | 871 | 570 |  |  |
|  | uvrR2153 | CTAATCTCDGTAAAAAATCCAACATAAG |  |  |  |  |
| *clpA* | clpA-F | AAAAACATCRAYYTTTTCATTTTTTAGTA | 783 | 570^*^ |  | [1] |
|  | clpA-R | TTGACYTATTAGATGGTCTTGG |  |  |  |  |
| *nifS* | nifS-F | GAAAMAKTMAAAATCMTAAGGAAAG | 861 | 564 |  |  |
|  | nifS-R | CAATAATTCCTGCAATGTTTGGTG |  |  |  |  |
| *16S* rRNA | 16S F | AGAGTTTGATCCTGG | ~1,500 |  | 95˚C for 5 min, 40 cycles of 95˚C for 30 s, 56˚C for 30 s, and 72˚C for 90 s, followed by a final extension at 72˚C for 10 min. | [2] |
|  | 16S R | TACCTTGTTACGACTT |  |  |  |  |
| *flaB* | BOR1 | TAATACGTCAGCCATAAATGC | ~770 |  |  | [3] |
|  | BOR2 | GCTCTTTGATCAGTTATCATTC |  |  |  |  |
| *glpQ* fragement 1 | glpQ-F1 | CATTAATTATAGCTCACAGAG | 599 |  |  | [4] |
|  | glpQ-R1 | AACAAGCATTATCAATTTTCC |  |  |  |  |
| *glpQ* fragement 2 | glpQ-F2 | TATGGCATAAACAACAAGGTA | 453 |  |  |  |
|  | glpQ-R2 | AATCTGTAAATAGACCATCTA |  |  |  |  |
| *16s*  *cytB* | mt-rrs-F  mt-rrs-R  *cytB*-F  *cytB*-R | CTGCTCAATGATTTTTTAAATTGCTGTGG  CCGGTCTGAACTCAGATCAAGTA  CCATGAGGCCAAATATCCTTCTGAGG  TTGGCCAATGATAATGTAKGGRTGTTC | 460 |  | 95˚C for 5 min, 40 cycles of 95˚C for 30 s, 55˚C for 30 s, and 72˚C for 90 s, followed by a final extension at 72˚C for 10 min. | [5]  [6] |

^*^Locus with unstandard size, the table showed the size of this study.

^#^The curator provided a set of primers that might work for a variety of relapsing fever borreliae, the table showed the primers that worked well with our sample.

**Reference**

1. Kingry LC, Anacker M, Pritt B, Bjork J, Respicio-Kingry L, Liu G, et al. Surveillance for and Discovery of *Borrelia* Species in US Patients Suspected of Tickborne Illness. Clin Infect Dis. 2018;66(12):1864-71. Epub 2017/12/23. doi: 10.1093/cid/cix1107. PubMed PMID: 29272385; PubMed Central PMCID: PMCPMC5985202.

2. Evans NJ, Brown JM, Demirkan I, Singh P, Getty B, Timofte D, et al. Association of unique, isolated treponemes with bovine digital dermatitis lesions. J Clin Microbiol. 2009;47(3):689-96. Epub 2009/01/16. doi: 10.1128/jcm.01914-08. PubMed PMID: 19144804; PubMed Central PMCID: PMCPMC2650952.

3. Assous MV, Wilamowski A, Bercovier H, Marva E. Molecular characterization of tickborne relapsing fever *Borrelia*, Israel. Emerg Infect Dis. 2006;12(11):1740-3. Epub 2007/02/08. doi: 10.3201/eid1211.060715. PubMed PMID: 17283626; PubMed Central PMCID: PMCPMC3372360.

4. Toledo A, Anda P, Escudero R, Larsson C, Bergstrom S, Benach JL. Phylogenetic analysis of a virulent *Borrelia* species isolated from patients with relapsing fever. J Clin Microbiol. 2010;48(7):2484-9. Epub 2010/05/14. doi: 10.1128/jcm.00541-10. PubMed PMID: 20463158; PubMed Central PMCID: PMCPMC2897527.

5. Takano A, Fujita H, Kadosaka T, Takahashi M, Yamauchi T, Ishiguro F, et al. Construction of a DNA database for ticks collected in Japan: application of molecular identification based on the mitochondrial 16S rDNA gene. Medical Entomology and Zoology. 2014;65(1):13-21. doi: 10.7601/mez.65.13.

6. Ishii A, Ueno K, Orba Y, Sasaki M, Moonga L, Hang'ombe BM, et al. A nairovirus isolated from African bats causes haemorrhagic gastroenteritis and severe hepatic disease in mice. Nat Commun. 2014;5:5651. Epub 2014/12/03. doi: 10.1038/ncomms6651. PubMed PMID: 25451856; PubMed Central PMCID: PMCPMC4268697.
