# Supplementary material for: Human-pathogenic relapsing fever Borrelia found in bats from Central China phylogenetically clustered together with relapsing fever borreliae reported in the New World
Source: PLoS Negl Trop Dis. 2021 Mar 18;15(3):e0009113. doi: 10.1371/journal.pntd.0009113 (PMC7971464; doi:10.1371/journal.pntd.0009113)
Supplement: S2 Table — (DOCX) [file pntd.0009113.s010.docx]

**S2 Table. Estimates of evolutionary divergence between relapsing fever *Borrelia* species based on the concatenated 8 housekeeping gene sequences (*clpA*-*clpX*-*nifS*-*pepX*-*pyrG*-*recG*-*rplB*-*uvrA*).**

|  | 1 | 2 | 3 | 4 | 5 | 6 | 7 | 8 | 9 | 10 | 11 | 12 | 13 | 14 | 15 |
| --- | --- | --- | --- | --- | --- | --- | --- | --- | --- | --- | --- | --- | --- | --- | --- |
| 1 *Borrelia* sp*.* (ST938) |  |  |  |  |  |  |  |  |  |  |  |  |  |  |  |
| 2 *Borrelia* sp*.* (ST927) | 0.004 |  |  |  |  |  |  |  |  |  |  |  |  |  |  |
| 3 *Candidatus* Borrelia fainii (LC365919-26)^*^ | 0.015 | 0.015 |  |  |  |  |  |  |  |  |  |  |  |  |  |
| 4 *Candidatus* Borrelia johnsonii (ST764) | 0.032 | 0.032 | 0.028 |  |  |  |  |  |  |  |  |  |  |  |  |
| 5  *Borrelia turicatae* (ST116) | 0.034 | 0.034 | 0.031 | 0.027 |  |  |  |  |  |  |  |  |  |  |  |
| 6 *Borrelia parke*ri (ST670) | 0.035 | 0.035 | 0.030 | 0.025 | 0.019 |  |  |  |  |  |  |  |  |  |  |
| 7 *Borrelia hermsii* (ST104) | 0.099 | 0.097 | 0.096 | 0.094 | 0.089 | 0.090 |  |  |  |  |  |  |  |  |  |
| 8 *Borrelia coriaceae* (ST671) | 0.112 | 0.112 | 0.109 | 0.107 | 0.104 | 0.105 | 0.113 |  |  |  |  |  |  |  |  |
| 9 *Borrelia anserina* (ST668) | 0.128 | 0.128 | 0.128 | 0.125 | 0.126 | 0.124 | 0.114 | 0.147 |  |  |  |  |  |  |  |
| 10 *Borrelia miyamotoi* (ST633) | 0.135 | 0.134 | 0.130 | 0.129 | 0.127 | 0.128 | 0.127 | 0.143 | 0.153 |  |  |  |  |  |  |
| 11 *Borrelia duttonii* (ST101) | 0.155 | 0.154 | 0.151 | 0.152 | 0.150 | 0.150 | 0.153 | 0.165 | 0.168 | 0.173 |  |  |  |  |  |
| 12 *Borrelia recurrentis* (ST669) | 0.155 | 0.154 | 0.150 | 0.151 | 0.150 | 0.150 | 0.152 | 0.164 | 0.168 | 0.173 | 0.003 |  |  |  |  |
| 13 *Borrelia crocidurae* (ST672) | 0.157 | 0.156 | 0.153 | 0.151 | 0.151 | 0.151 | 0.152 | 0.165 | 0.169 | 0.175 | 0.007 | 0.006 |  |  |  |
| 14 *Borrelia persica* (ST636) | 0.157 | 0.156 | 0.154 | 0.155 | 0.156 | 0.151 | 0.156 | 0.168 | 0.178 | 0.177 | 0.113 | 0.113 | 0.113 |  |  |
| 15 *Borrelia tanukii* (ST454) | 0.296 | 0.294 | 0.295 | 0.296 | 0.294 | 0.292 | 0.301 | 0.298 | 0.307 | 0.314 | 0.281 | 0.281 | 0.281 | 0.292 |  |

Estimates of evolutionary divergence between relapsing fever *Borrelia* species based on the concatenated 8 housekeeping gene sequences (*clpA*-*clpX*-*nifS*-*pepX*-*pyrG*-*recG*-*rplB*-*uvrA*). Analyses were conducted using the Kimura 2-parameter model in MEGA7. *Candidatus* Borrelia fainii was not assigned with a ST number, because the 8 sequences were not submitted to the *Borrelia* MLST database (https://pubmlst.org/borrelia/). * indicated that GenBank accession number instead of ST number was shown.
